# Prevalence and risk factors of elopement behavior in children and adolescents with autism spectrum disorder: a systematic review and meta-analysis

**DOI:** 10.3389/fped.2026.1957224

**Published:** 2026-09-30

**Authors:** Lai Ciai, Huang Guolin, Chen Xi, Li Rongzhen, Lian Xionghan, Li Xin, Cai Aiyuan

**Affiliations:** 1 Department of Pediatrics, Shenzhen Hospital (Futian) of Guangzhou University of Traditional Chinese Medicine, Shenzhen, China; 2 Department of Pediatrics, Maoming Maternal and Child Health Hospital, Maoming, China; 3 Department of Pediatrics, Longhua District People's Hospital, Shenzhen, Guangdong, China

**Keywords:** autism spectrum disorder, challenging behavior, elopement behavior, meta-analysis, risk factors, wandering away

## Abstract

**Objective:**

Wandering and elopement are common and potentially life-threatening behaviors in children and adolescents with autism spectrum disorder (ASD). Despite extensive research, debates persist regarding the chronic nature of these behaviors, their relationship with cognitive and linguistic functions, and the primary risk factors. This study aims to quantitatively assess the incidence of elopement in this population and systematically evaluate associated risk factors.

**Methods:**

Systematic searches were conducted in PubMed, Embase, Web of Science, and Cochrane Library databases to gather observational studies on the incidence and risk factors of elopement in individuals with ASD (≤18 years old) from the inception of the databases to August 2026. Data were pooled using random or fixed-effect models, and subgroup analyses were performed. This study is registered in PROSPERO (CRD420261456297).

**Results:**

Fourteen observational studies were included. Meta-analysis revealed that the pooled incidence of elopement among children with ASD was 46% (95% CI: 36%–56%). Subgroup analysis showed no significant difference between recent (43%) and lifetime incidence (51%) (*P* = 0.4954), highlighting the chronic nature of this behavior. Additionally, no significant differences were found in elopement risk across genders (40% in males vs. 31% in females, *P* = 0.4374) or cognitive/linguistic function levels (41% in severe/ID/non-verbal group vs. 45% in mild/without ID/verbal group, *P* = 0.8434). Risk factor analysis identified comorbid attention-deficit/hyperactivity disorder (ADHD, aOR = 3.00), lack of danger awareness (aOR = 2.20), and communication disorders (OR = 2.24) as highly correlated with elopement behavior.

**Conclusion:**

Elopement is a prevalent and chronic behavior in children with ASD, unaffected by improvements in cognitive or linguistic abilities, challenging traditional clinical assumptions. In clinical practice, elopement prevention should be part of routine assessments, with interventions focusing on managing comorbidities like ADHD and enhancing safety skills training.

## 1 Introduction

Autism spectrum disorder (ASD) is a complex neurodevelopmental condition characterized by core social and behavioral deficits. In addition to these, children with ASD often display challenging behaviors, such as aggression and self-injury, which significantly impact their quality of life and that of their families (1). These behaviors present urgent challenges for traditional clinical interventions like applied behavior analysis (2). However, as research advances, the focus of clinical intervention is shifting. Ferina et al. emphasize that effectively predicting and managing these challenging behaviors are now central goals for enhancing patient safety and optimizing intervention strategies (3). Among these behaviors, elopement or wandering is particularly concerning as it poses a major public health risk, often leading to serious accidents. Recent data on missing persons reveal that the mortality rate for children with ASD who go missing is 19.2%, with over 90% of accidental deaths due to drowning (4). It remains unclear whether this high-risk behavior is a transient developmental phase or a persistent challenge throughout childhood.

When assessing challenging behaviors in ASD, there is significant variability in patients’ cognitive and language functions. Recently, the Lancet Commission and Siegel et al. have identified a subgroup termed “profound autism,” characterized by severe intellectual disabilities and a need for constant adult support (5, 6). This subgroup is generally associated with a higher incidence of serious challenging behaviors. However, the relationship between elopement and cognitive/language levels is still debated. While some studies suggest that severe cognitive deficits and communication disorders are key predictors of elopement (7, 8), a recent large-scale national epidemiological survey presents a different perspective. The survey indicates that high-functioning children with ASD, who possess better motor and language skills, also face significant elopement risks. The incidence rate of 32.7% is statistically similar to that of children with severe intellectual disabilities (9). This finding suggests that elopement is a widespread risk across the autism spectrum, not limited to profound autism. High-functioning children may elope due to goal-oriented motivations or to escape sensory overload, challenging traditional risk assessment methods that focus solely on cognitive levels (10). Furthermore, the exact causes of elopement remain unknown, and the potential influence of comorbid mental disorders, such as ADHD or anxiety, warrants systematic evaluation.

Despite the significant clinical risks associated with elopement behavior, no quantitative meta-analysis has systematically summarized its incidence and related risk factors. This study aims to address this gap. Our primary objectives are: (1) to quantitatively assess the overall incidence of elopement in children with ASD and compare different time windows to explore its chronicity; (2) to examine incidence differences among cognitive, language, and gender subgroups within the “severe autism” and mild/high-functioning autism frameworks; and (3) to identify risk factors significantly correlated with elopement behavior, thereby providing an evidence-based foundation for developing targeted intervention strategies to prevent elopement.

## 2 Methods

This systematic review and meta-analysis were crafted following the Preferred Reporting Items for Systematic Reviews and Meta-Analyses (PRISMA 2020) guidelines. It was pre-registered in the PROSPERO international prospective systematic review system under registration number CRD420261456297.

### 2.1 Search strategy

Two researchers independently performed an extensive computer search across four major core databases: PubMed, Embase, Web of Science (Core Collection), and the Cochrane Library. The search covered the period from the inception of these databases up to August 20, 2026. To maximize the recall rate, the search strategy incorporated both subject terms (such as MeSH and Emtree) and free-text terms. Key English search terms included “autism spectrum disorder,” “Autism,” “ASD,” “Wandering Behavior,” “elopement,” “running away,” and “absconding.” Additionally, we broadened the search to encompass related terms for high-risk behaviors, such as “high risk behavior” and “challenging behavior,” to ensure comprehensive literature capture. Furthermore, we manually reviewed the reference lists of the included studies and relevant reviews to identify additional eligible studies.

### 2.2 Eligibility criteria

This study's literature screening follows the PICOS principle. The population (P) focuses on children and adolescents, typically aged 18 years or younger, diagnosed with ASD using established clinical criteria such as the DSM-5, ICD-10, or ADOS. Studies centered on adult ASD patients or those failing to clearly differentiate ASD from other neurodevelopmental disorders, like intellectual disability, are excluded. For intervention/exposure (I/E), the study includes research that explicitly documents behaviors such as “wandering,” “elopement,” “running away,” or “absconding from home/school” in children with ASD. The control group (C) consists of typically developing children or those with other conditions, such as attention-deficit/hyperactivity disorder (ADHD) or intellectual disability (ID) without a diagnosis of ASD. In single-arm epidemiological studies that only report incidence rates, a control group is not required. Outcome measures (O) include the primary outcome of pooled prevalence (%) of wandering/elopement behaviors. Secondary outcomes involve the incidence of accidental injuries, such as traffic accidents or drowning, resulting from elopement, and the identification of risk factors like intellectual level, disease severity, and comorbidity, with their Odds Ratio (OR) or Relative Risk (RR) extracted. The study design (S) encompasses observational studies, including cross-sectional, cohort, and case-control studies. Excluded are reviews, case reports, qualitative studies, conference abstracts lacking complete data, and non-human animal experiments.

### 2.3 Data extraction and quality assessment

Two researchers independently extracted data from the selected literature. Each study underwent an independent review to ensure methodological adequacy, focusing on clear diagnostic criteria for autism spectrum disorder (ASD) and a precise definition of wandering. If disagreements arose during data extraction, the researchers discussed them; if unresolved, a third senior researcher was consulted for a decision. The extracted data included: (1) Basic Study Characteristics: Information such as the first author's name, publication year, the study's country or region, and the study design type (e.g., cross-sectional, cohort). (2) Demographic and Clinical Characteristics: Details on sample size, age range or mean age, gender ratio, and diagnostic criteria or assessment tools for ASD. (3) Outcome Indicators: Data on the incidence of wandering or elopement behavior, including definitions for lifetime and recent incidence, as well as incidence data for different cognitive and language function subgroups. (4) Risk Factor Data: Effect size indicators for potential risk factors associated with elopement behavior, such as comorbid ADHD, lack of danger awareness, and communication disorders. This included odds ratios (OR) or adjusted odds ratios (aOR) and their 95% confidence intervals (CI). For studies reporting only incidence percentages without specific event numbers, we calculated the number of elopements by multiplying the total sample size by the percentage and rounding off. When subgroup data or 95% CIs were not directly reported, we estimated them using other raw data provided, such as totals, regression coefficients, and standard errors, whenever possible.

### 2.4 Statistical analysis

All statistical analyses were performed using the “meta” package in R (version 4.4.1). For the primary outcome, the incidence of wandering, the Logit transformation (PLOGIT) was applied to the raw data to stabilize variance and maintain confidence intervals within the 0 to 1 range. The Inverse Variance method was then employed for quantitative synthesis. To evaluate statistical heterogeneity among the studies, Cochran's *Q* test and the I^2^ statistic were used. If I^2^ exceeded 50% or P was less than 0.10, indicating significant heterogeneity, a Random-effects model was adopted, using the restricted maximum likelihood method (REML) to estimate between-group variance (tau^2^). Otherwise, a Fixed-effects model was applied. Subgroup analyses were pre-set and conducted to explore potential sources of heterogeneity in overall incidence, focusing on the assessment time window (lifetime vs. recent), gender (male vs. female), and cognitive/linguistic function levels. For the secondary outcome, which involved risk factors, odds ratios (OR) or relative risks (RR) and their 95% confidence intervals (CI) were extracted from each study. The standard error (SE) was calculated using the natural logarithm transformation (logOR), and a forest plot was created for a visual summary. Due to inherent clinical heterogeneity among different risk factors, the study provided an overview of effect sizes without conducting an overall pooling that would lack clinical significance. Sensitivity analysis was performed using the Leave-one-out method to verify the robustness of the pooled incidence results. Following the Cochrane Handbook for Systematic Reviews of Interventions, since fewer than 10 studies were included in the final quantitative synthesis, a Funnel plot or Egger's test was not formally used to assess publication bias, as this number is below the threshold needed to ensure sufficient statistical power to detect asymmetry.

## 3 Results

### 3.1 Results of literature retrieval and screening

Initially, 3,084 relevant articles were retrieved from four major core databases. After removing 1,056 duplicates using literature management software, 2,028 articles remained. Titles and abstracts were independently reviewed for initial screening, resulting in the exclusion of 1,967 articles that did not meet the inclusion criteria. This left 61 articles for full-text eligibility assessment. During this stage, 47 articles were excluded due to various reasons: 27 had inappropriate study designs, 10 lacked relevant outcome indicators, 5 involved unsuitable study populations, and 5 had overlapping populations. Ultimately, 14 observational studies were included in this systematic review. Of these, data from 13 articles were used for quantitative meta-analysis, with 8 contributing to the overall incidence pooling and 7 to the risk factors pooling. One article (Andersen et al., 2020) was excluded from quantitative pooling due to overlapping study populations with an existing database; it was utilized solely for narrative synthesis of high-frequency wandering characteristics and intervention measures. The entire literature search and screening process adhered to the PRISMA 2020 guidelines, as depicted in Figure 1.

**Figure 1:**
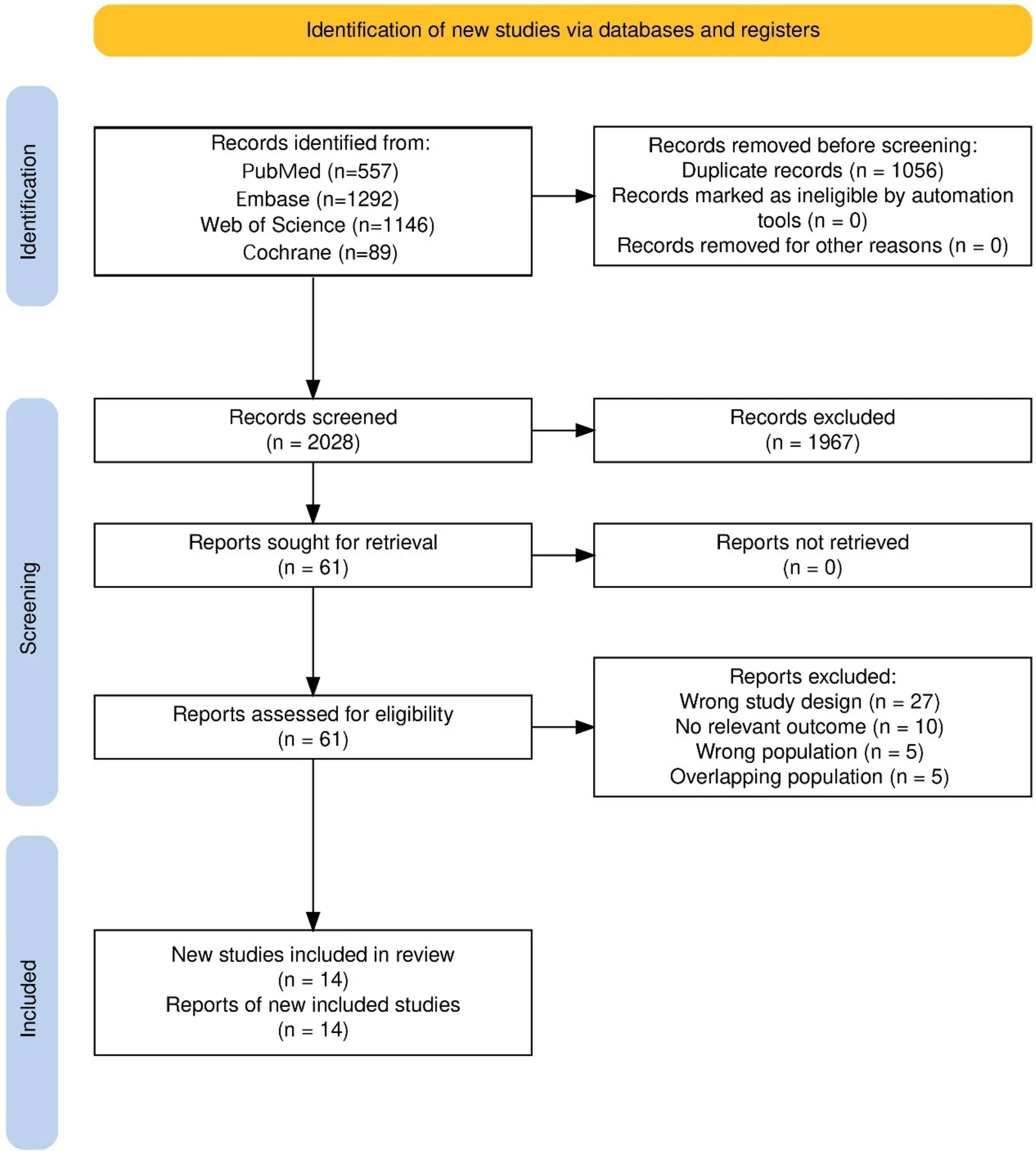
Prisma 2020 flow diagram of the literature search and study selection process.

### 3.2 Basic characteristics of the included studies and handling of population overlap

Fourteen observational studies, published between 2011 and 2026, were analyzed, encompassing regions such as the United States, Australia, and Iraq. The sample sizes varied from 84 to 1774 participants, with subjects’ ages ranging from preschool (2 years old) to adulthood. Notably, several studies utilized the same large-scale national cohort databases. For instance, Rice et al. (2016), Kiely et al. (2016), and Barnard-Brak (2016) all drew from the “Pathways to ASD” cohort in the United States. Similarly, Wiggins et al. (2020, 2026) used the “SEED” cohort, while Anderson et al. (2012), Vasa et al. (2019), and Andersen et al. (2020) sourced data from the “IAN Registry” database. To maintain the independence assumption crucial for meta-analysis and prevent double-counting, only the study with the largest sample size or most comprehensive data from each database was selected for pooling the “overall incidence.” This approach resulted in the inclusion of eight independent studies, with a cumulative sample size of 5552. Studies with overlapping populations were utilized solely for extracting uniquely reported risk factors (OR/aPR values) or specific subgroup characteristics. Table 1 provides detailed baseline characteristics of the included studies and outlines the data extraction purposes.

**Table 1:** Basic characteristics and data extraction of the included literatures.

Study	Year	Country	Study design	Age range	Database/cohort	Sample size (N)	Assessment window	Data extracted for meta-analysis
Hattier et al. (11)	2011	USA	Cross-sectional	17–37 months	EarlySteps Programme	633	Current	Prevalence
Jang et al. (12)	2011	USA	Cross-sectional	29–218 months	CARD Clinic	84	Current	Prevalence
Anderson et al. (7)	2012	USA	Cross-sectional	4–17 years	IAN Registry	1218	Lifetime	Prevalence, Risk Factors
Barnard-Brak (13)	2016	USA	Cross-sectional	6–17 years	Pathways to ASD	1774	Past 12 Months	Risk Factors (Independent ORs)
Kiely et al. (10)	2016	USA	Cross-sectional	6–17 years	Pathways to ASD	1416	Past 12 Months	Risk Factors (Behavioral ORs)
Rice et al. (9)	2016	USA	Cross-sectional	6–17 years	Pathways to ASD	1420	Past 12 Months	Prevalence, Risk Factors
Pereira-Smith (14)	2019	USA	Cross-sectional	2–17 years	MUSC Clinic	394	Lifetime	Prevalence, Risk Factors
Vasa et al. (15)	2020	USA	Cross-sectional	3–25 years	IAN Registry	462	Past 3 Months	Risk Factors (Consequences)
Andersen et al. (8)	2020	USA	Cross-sectional	≥ 4 years	IAN Registry	526	Past 2 Years	Risk Factors (High-Frequency EB), Interventions
Wiggins et al. (16)	2020	USA	Cross-sectional	4–5 years	SEED Cohort	1195	Past 3 Months	Prevalence, Risk Factors
DiGuiseppi et al. (17)	2025	USA	Cross-sectional	30–68 months	SEED Cohort	648	Past 6 Months	Risk Factors (Consequences)
Hodge et al. (18)	2025	Australia	Cross-sectional	1.5–16.7 years	Sydney Child Neurodevelopment Registry	507	Current	Prevalence, Risk Factors
Samadi & Rashid (19)	2025	Iraq	Cross-sectional	3–19 years	Daycare Center	101	Lifetime	Prevalence, Risk Factors
Wiggins et al. (20)	2026	USA	Longitudinal	2–5y & 12-16y	SEED Cohort	258	Past 3 & 12 Months	Risk Factors (Longitudinal aPR)

To avoid double-counting, for studies utilizing the same database such as Pathways to ASD or SEED, only one representative study was included in the overall prevalence pooling. The overlapping studies were exclusively utilized for extracting unique risk factors or subgroup analyses.

IAN, Interactive Autism Network; SEED, Study to Explore Early Development.

### 3.3 Overall prevalence of wandering and getting lost

Eight cross-sectional studies, encompassing a total of 5,552 children and adolescents with ASD, provided data on the incidence of elopement/wandering, which were pooled for analysis. Given the substantial heterogeneity among these studies (I^2^ = 97.9%, *p* < 0.0001), a random-effects model was employed for the meta-analysis. The forest plot results (Figure 2A) indicated that the overall pooled incidence of elopement/wandering behavior in the ASD population was 46% (95% CI: 36%—56%). To evaluate the stability of these findings, a leave-one-out meta-analysis was conducted. This analysis (Figure 2B) revealed that the pooled incidence remained consistent, ranging from 42% (when excluding Wiggins et al. 2020) to 50% (when excluding Rice et al. 2016), underscoring the robustness of the overall pooled results.

**Figure 2:**
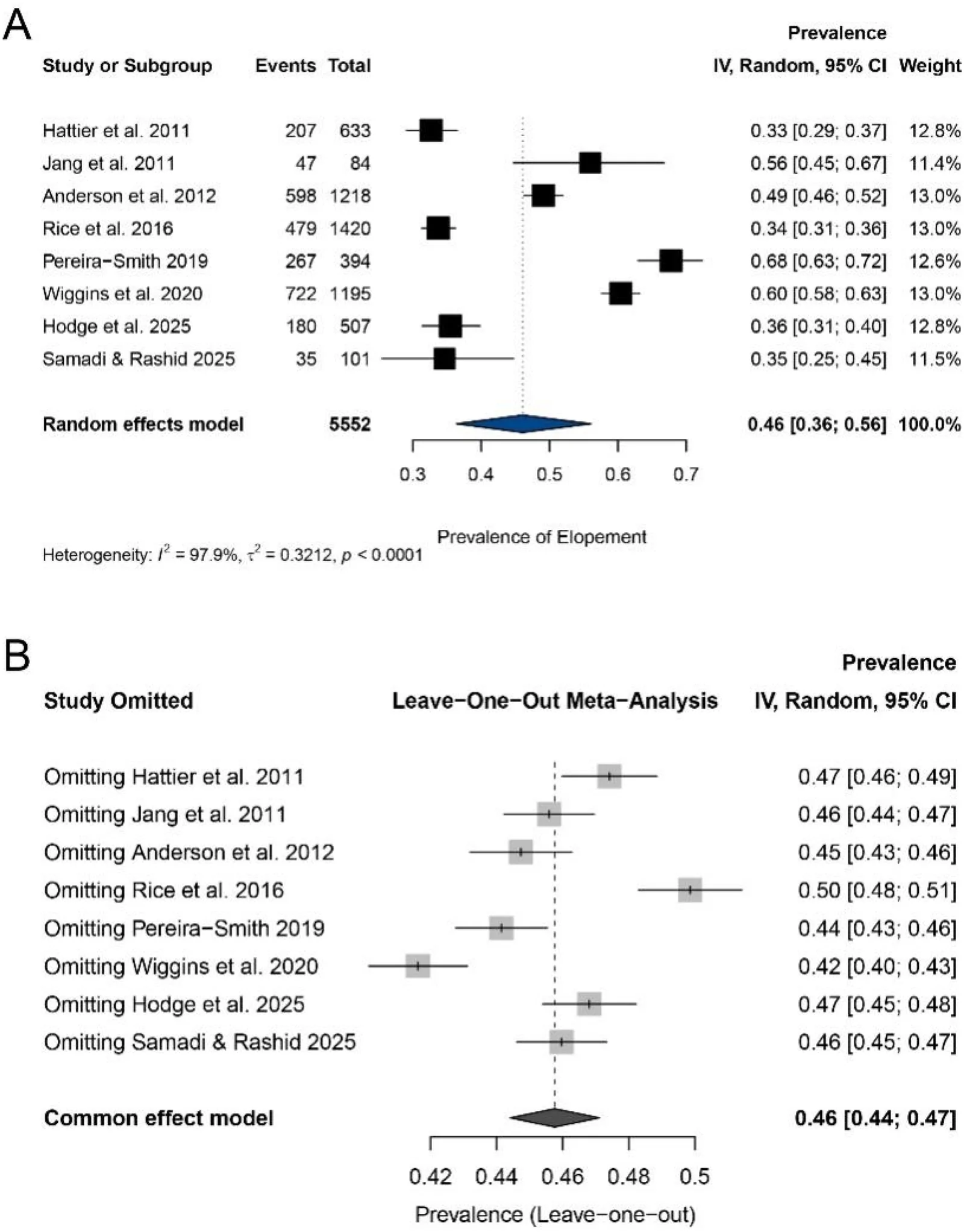
Meta-analysis of the overall prevalence of elopement/wandering behavior in children and adolescents with ASD. **(A)** Forest plot of the pooled prevalence using a random-effects model. **(B)** Leave-one-out sensitivity analysis demonstrating the robustness of the pooled prevalence.

### 3.4 Subgroup analysis

To investigate the causes of the significant variability in overall prevalence, we performed pre-specified subgroup analyses:

1. Stratified by assessment window:

during the data extraction phase, we carefully documented the assessment time windows for each original study (see Table 1 for details). To enhance the statistical power of our subgroup analysis and explore clinical significance, we re-categorized the time windows into “Lifetime” and “Recent” for the meta-analysis. “Lifetime” refers to instances of elopement or wandering behavior occurring at any point from childhood in children with autism. In contrast, “Recent” encompasses studies conducted at present or with a defined short-term retrospective period, such as the past 3 or 12 months, reflecting current behavioral characteristics. Based on these assessment time windows, the eight included incidence studies were divided into two subgroups: “Recent” (5 studies) and “Lifetime” (3 studies) (Figure 3). Our findings revealed that the pooled incidence of lifetime elopement/wandering was 51% (95% CI: 32%–69%), while the recent incidence was 43% (95% CI: 32%–55%). The subgroup difference test showed no statistically significant difference between them (*p* = 0.4954). This suggests that elopement and wandering are prevalent in the ASD population, regardless of whether they are considered historical behaviors or current states.

2. Stratified by gender:

**Figure 3:**
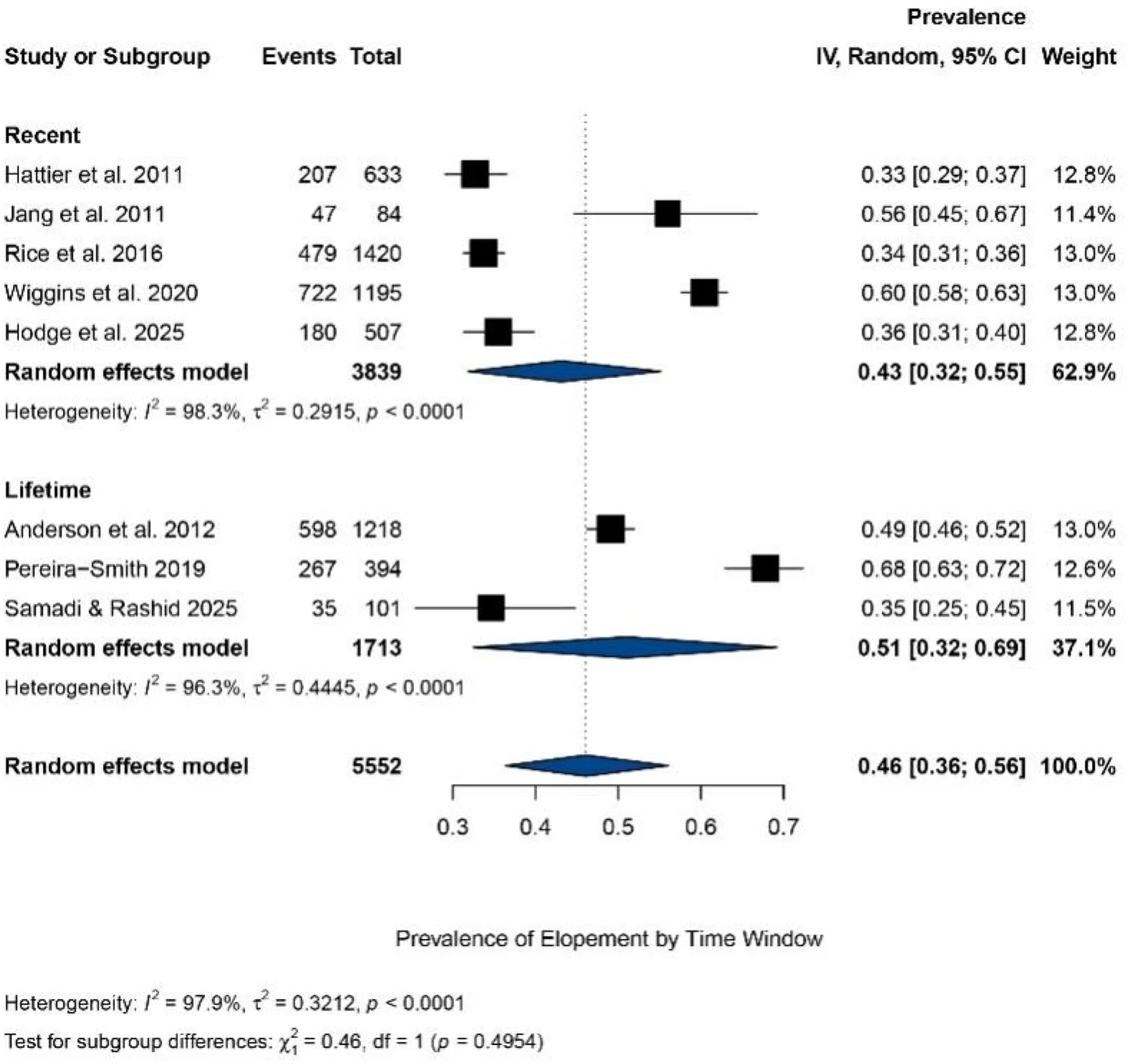
presents a forest plot of the subgroup analysis for the prevalence of elopement based on the assessment window (recent vs. lifetime).

To assess how gender influences the incidence of elopement/wandering behavior, we performed a subgroup analysis on 5 studies, encompassing 6 independent datasets with a total of 3,222 patients, all of which provided clear gender data (Figure 4). The analysis revealed that the pooled incidence of elopement/wandering in male children with ASD was 40% (95% CI: 25%–57%, *N* = 2,275), compared to 31% (95% CI: 19%–47%, *N* = 947) in female children. Despite the slightly higher incidence in males, the subgroup difference test showed no statistically significant difference between genders (*p* = 0.4374).

1. Classification based on cognitive and linguistic functions:

**Figure 4:**
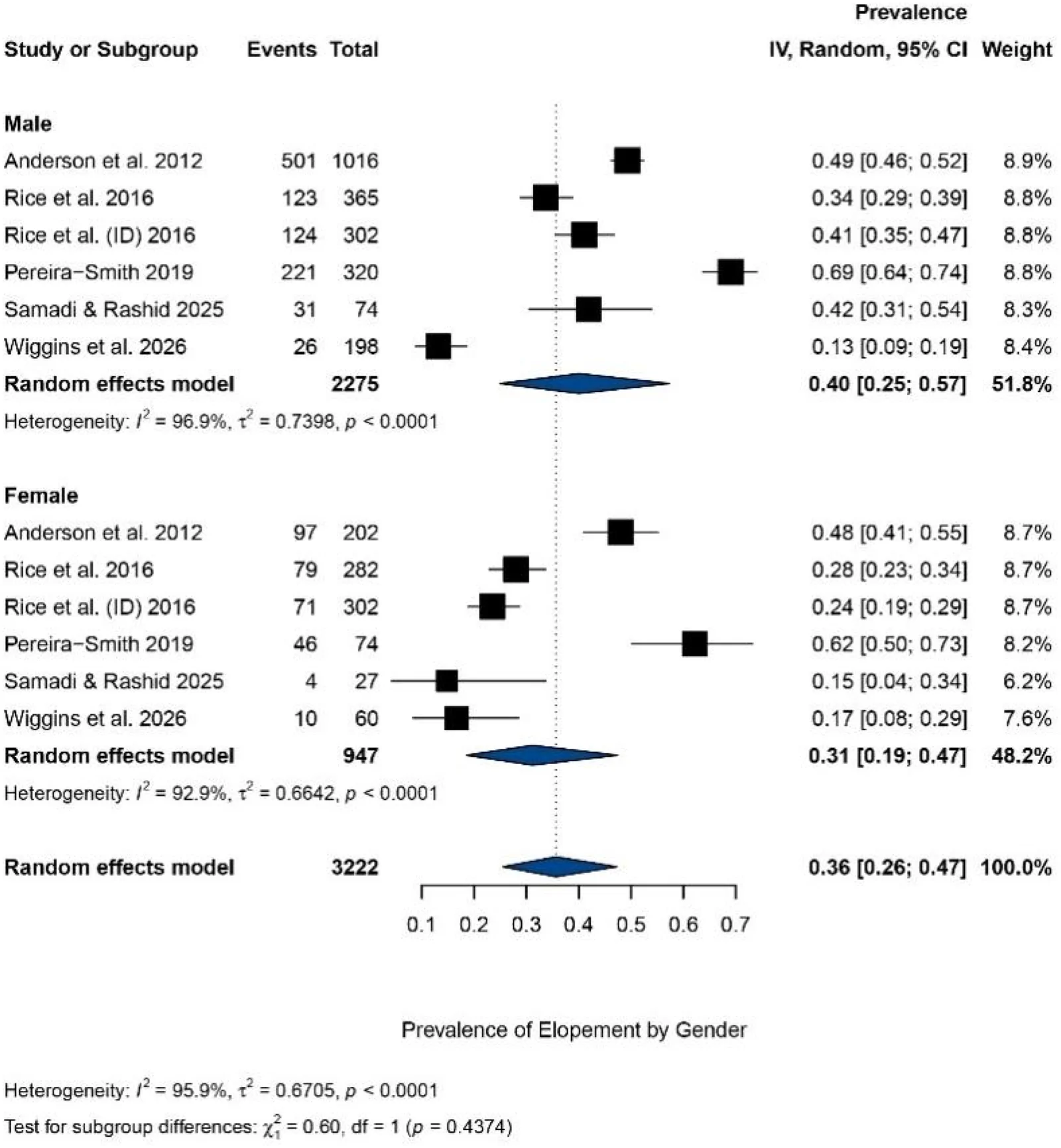
presents a forest plot of the subgroup analysis for the prevalence of elopement based on gender (male vs. female).

To assess whether children's cognitive and language abilities impact the risk of wandering or becoming lost, we analyzed data from five studies, encompassing a total of 3,618 patients. Due to varying classification criteria across these studies, we categorized the children into two subgroups: “Severe or ID or Non-verbal” and “Mild or No ID or Verbal” (Figure 5). Our analysis revealed that the pooled incidence of wandering/loss among children with ASD who were severe, had an intellectual disability (ID), or were non-verbal was 41% (95% CI: 17%–71%, *N* = 1,047). In contrast, for children who were mild, had no ID, or were verbal, the incidence was 45% (95% CI: 33%–57%, *N* = 2,571). The subgroup difference test results showed no statistically significant difference in the incidence of wandering/loss between children with varying cognitive and language function levels (*p* = 0.8434).

**Figure 5:**
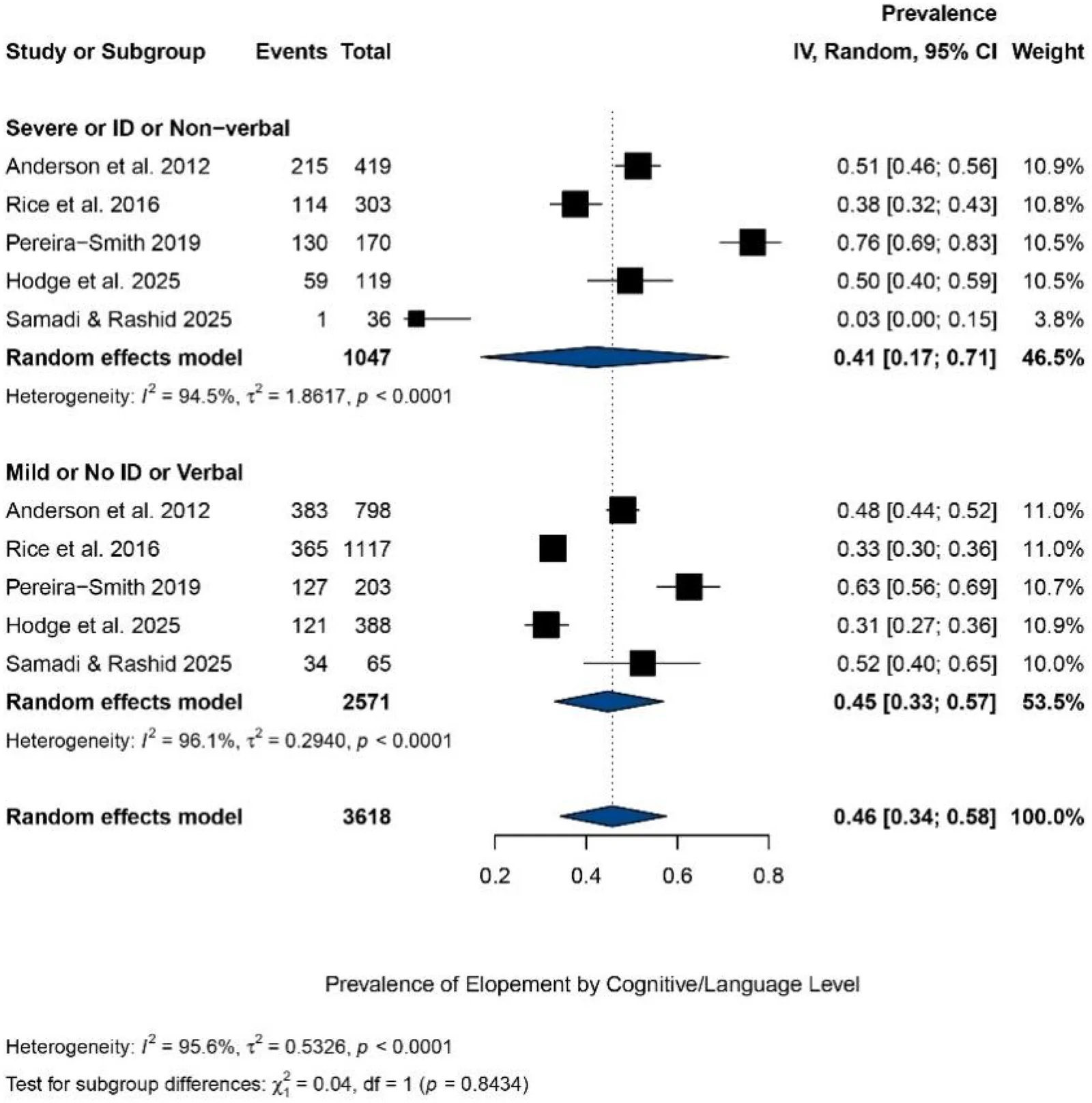
presents a forest plot of the subgroup analysis for the prevalence of elopement based on cognitive/language level (severe or ID or non-verbal vs. mild or no ID or verbal).

### 3.5 Risk factors for wandering away

Several studies have investigated potential risk factors for wandering and elopement behaviors in children with ASD. Given the diverse predictors examined across these studies, we have visually summarized the significant risk factors using a forest plot (Figure 6). The analysis revealed that all identified factors notably increased the risk of elopement, as indicated by effect sizes greater than 1 and 95% confidence intervals excluding 1. Notably, comorbid ADHD emerged as the strongest independent risk factor for elopement, with an aOR of 3.00 (95% CI: 2.30–3.91). This was followed by comorbid anxiety disorder, which had an aOR of 1.63 (95% CI: 1.24–2.14). Additionally, both Lack of Danger Awareness (aOR = 2.20) and Limited Communication (OR = 2.24) more than doubled the risk of elopement. Other significant predictors included a diagnosis of ASD (OR = 1.23), Profound Autism (OR = 2.17), and overall symptom severity (RR = 1.09; OR = 1.27).

**Figure 6:**
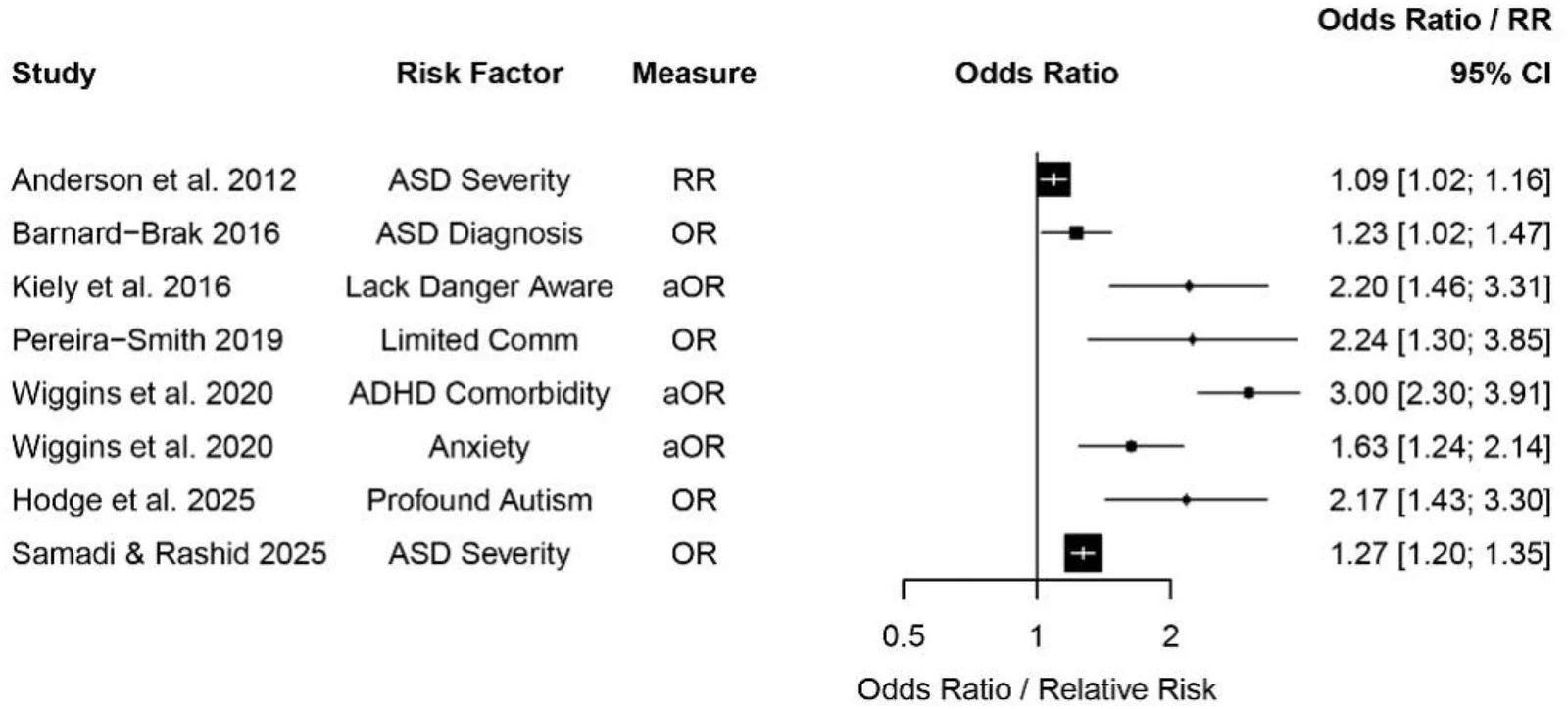
presents a forest plot of the meta-analysis for the risk factors significantly associated with elopement in ASD, showing pooled effect sizes (ORs/RRs and 95% CIs).

## 4 Discussion

This study is the first quantitative meta-analysis examining the incidence and risk factors associated with wandering and elopement behaviors in children with ASD. Our findings reveal that elopement is a common and challenging behavior within the ASD population, with a pooled incidence rate of 46%. Notably, this study challenges traditional clinical assumptions by showing no significant differences in elopement risk based on gender, cognitive abilities, or language levels. However, we found strong associations between elopement behavior and comorbid conditions such as ADHD, lack of danger awareness, and communication disorders. In the following sections, we will delve into the potential mechanisms underlying these findings and discuss their significant implications for clinical care within existing frameworks.

Our time-window subgroup analysis directly addresses the introductory question of whether elopement behavior is a transient phenomenon. The analysis reveals no significant statistical difference between the recent incidence rate of 43% and the lifetime incidence rate of 51%. As noted in existing studies, while the lifetime incidence rate is inherently higher than the recent incidence rate, the behaviors of children with ASD can vary across developmental stages (7). Despite these fluctuations, both incidence rates in our study remain consistently high, exceeding 40%. This finding indicates that elopement behavior is not just a temporary deviation during a specific developmental stage but rather a persistent issue with chronic characteristics. This conclusion aligns closely with a recent narrative review on the trajectory and prediction of challenging behaviors in ASD (3). Consequently, screening for elopement risk should not be a one-time event; instead, it should be integrated into routine clinical care, involving ongoing longitudinal assessments.

The most clinically significant finding in this study emerges from the cognitive and language function subgroups. Profound autism is typically associated with severe intellectual disabilities, minimal or no language skills, and a need for continuous care, often linked to a higher incidence of challenging behaviors (5, 6). Contrary to these expectations, our data reveal that the risk of elopement is similarly high for children with mild autism who have language abilities (45%) and those with profound autism who lack language abilities (41%). We introduce the “Supervision Paradox” to explain this unexpected result. Children with profound autism are generally under near-constant physical supervision due to their extreme vulnerability. The study indicates that caregivers frequently employ high-intensity response blocking and retrieval strategies to prevent high-risk behaviors (21). This constant state of high alert, necessary for managing sudden crises, imposes significant psychological stress and caregiving burdens (22). However, this rigorous supervision inadvertently limits the opportunities for successful elopement. In contrast, children with higher functioning, who possess better language and motor skills, are often granted more independence (23, 24). Despite this, they may lack an inherent awareness of dangers such as traffic or water, as indicated by the “lack of danger awareness” in Figure 6 (aOR = 2.20). This mismatch between “high independence” and “low danger awareness” renders them equally vulnerable to hazardous situations.

Our analysis challenges the traditional stereotype that “male children with ASD are at a higher risk of wandering off.” The data reveal no significant statistical difference in risk between female (31%) and male (40%) children. Modern psychiatric studies consistently show that female children with ASD often possess stronger camouflaging abilities, enabling them to mimic neurotypical behaviors in social settings to conceal their own challenges (25, 26). While this ability aids in social integration, it also obscures their underlying lack of safety awareness, leading caregivers to underestimate their actual safety risks. This finding underscores the necessity of eliminating gender bias when developing strategies to prevent wandering.

A comprehensive analysis of risk factors highlights the significant role of mental comorbidities in elopement. The study identified comorbid ADHD as the most potent independent risk factor (aOR = 3.00). Previous neuropsychological and clinical management research indicates that the coexistence of ASD and ADHD is not merely an additive effect of symptoms but results in more severe impairments in executive function and behavioral control (27). The combination of ASD's cognitive rigidity with ADHD's core symptoms, such as high impulsivity and inhibitory control disorders, substantially diminishes an individual's risk assessment and self-control abilities. This dual pathological mechanism can easily lead children to abruptly leave a caregiver's supervision without warning when triggered by external stimuli or internal emotional fluctuations. This insight suggests that clinicians should not view wandering and elopement as mere behavioral issues. Actively addressing relevant mental comorbidities, such as implementing specialized drug or behavioral interventions for ADHD symptoms in children with ASD, can enhance their executive function and serve as a crucial strategy for reducing the risk of sudden high-risk behaviors like elopement (28, 29).

Wandering is a common behavioral issue and a persistent challenge that places significant physical and mental strain on families of children with ASD (7). The “occurrence” and “high-frequency” of wandering may be influenced by distinct mechanisms. In a study based on the IAN cohort, Andersen et al. found that severe communication disorders and aggressive behaviors significantly elevate the risk of frequent wandering in children (8). While our meta-analysis primarily identified clinical risk factors, the consistently high incidence of wandering behavior underscores the substantial shortcomings in existing prevention interventions. Traditional physical barriers, such as door locks, offer limited effectiveness as they are passive measures that restrict children's freedom. Most parents find commercial GPS tracking devices merely “adequate,” citing high daily maintenance, like frequent charging, and children's reluctance to wear them. Additionally, psychiatric medications show minimal effectiveness and present notable side effects. This scenario underscores a significant gap in current clinical management. Future research should prioritize interdisciplinary collaboration in both research and engineering to create intelligent, proactive, and low-burden systems for predicting wandering and enabling non-invasive monitoring (30). Such advancements could significantly alleviate caregiver stress and enhance the safety of children.

While this study offers significant evidence-based insights, it is not without limitations. Primarily, the majority of the included literature consists of cross-sectional studies, which are inadequate for establishing causal relationships between risk factors and wandering behavior. Secondly, in the assessment of risk factors, we combined cognitive and communication deficits into broader categories, such as severe or moderate, because existing literature often treats them collectively, typically under “severe autism.” We recommend that future research investigate cognitive and communication statuses separately to explore their independent associations with elopement and other challenging behaviors. Thirdly, our study's quantitative analysis primarily focused on patients’ intrinsic clinical characteristics. However, complex external environments, like noisy supermarkets or crowded queuing areas, often impose sensory stress on children with ASD, stimulating their motivation to escape and acting as key catalysts for elopement (31). These external environmental factors warrant systematic evaluation in future research. Additionally, most high-quality data originate from Western developed countries, with a notable scarcity of data from low- and middle-income countries. This imbalance may restrict the global applicability of the findings. Lastly, variations in the definitions and assessment tools for “wandering” across studies contribute to considerable heterogeneity in the pooled incidence rate.

## 5 Conclusions

This study reveals that wandering and elopement are prevalent, with a combined incidence of 46%, and represent chronic challenging behaviors among children with ASD. Importantly, the risk of elopement does not significantly differ across genders or cognitive/linguistic function levels, challenging traditional clinical stereotypes. Additionally, comorbid ADHD, lack of danger awareness, and communication disorders are strongly correlated with elopement behavior. Consequently, elopement prevention should not rely solely on physical restraints, nor should intelligence or language levels be the sole criteria for risk assessment. Clinical practice should integrate elopement risk assessment into routine ASD care, emphasizing the screening and management of relevant psychiatric comorbidities and providing targeted safety awareness training for children. Future research should focus on exploring how external environmental triggers influence wandering behavior. Moreover, to address the limitations of existing physical prevention and tracking devices, there is a need to develop more intelligent, low-burden, and non-invasive monitoring technologies. Such advancements would effectively reduce caregiving pressures on families and comprehensively ensure children's safety.

## Data Availability

The original contributions presented in the study are included in the article/Supplementary Material, further inquiries can be directed to the corresponding author.
